# Segmental Duplication Implicated in the Genesis of Inversion 2*Rj* of *Anopheles gambiae*

**DOI:** 10.1371/journal.pone.0000849

**Published:** 2007-09-05

**Authors:** Mamadou B. Coulibaly, Neil F. Lobo, Meagan C. Fitzpatrick, Marcia Kern, Olga Grushko, Daniel V. Thaner, Sékou F. Traoré, Frank H. Collins, Nora J. Besansky

**Affiliations:** 1 Center for Global Health and Infectious Diseases, Department of Biological Sciences, University of Notre Dame, Notre Dame, Indiana, United States of America; 2 Malaria Research and Training Center, University of Bamako, Bamako, Mali; Texas A&M University, United States of America

## Abstract

The malaria vector *Anopheles gambiae* maintains high levels of inversion polymorphism that facilitate its exploitation of diverse ecological settings across tropical Africa. Molecular characterization of inversion breakpoints is a first step toward understanding the processes that generate and maintain inversions. Here we focused on inversion 2Rj because of its association with the assortatively mating Bamako chromosomal form of *An. gambiae,* whose distinctive breeding sites are rock pools beside the Niger River in Mali and Guinea. Sequence and computational analysis of 2Rj revealed the same 14.6 kb insertion between both breakpoints, which occurred near but not within predicted genes. Each insertion consists of 5.3 kb terminal inverted repeat arms separated by a 4 kb spacer. The insertions lack coding capacity, and are comprised of degraded remnants of repetitive sequences including class I and II transposable elements. Because of their large size and patchwork composition, and as no other instances of these insertions were identified in the *An. gambiae* genome, they do not appear to be transposable elements. The 14.6 kb modules inserted at both *2Rj* breakpoint junctions represent low copy repeats (LCRs, also called segmental duplications) that are strongly implicated in the recent (∼0.4N_e_ generations) origin of *2Rj*. The LCRs contribute to further genome instability, as demonstrated by an imprecise excision event at the proximal breakpoint of *2Rj* in field isolates.

## Introduction

Seventy years ago, Dobzhansky expressed “…little doubt that chromosomal changes are one of the mainsprings of evolution” [1]. Although this view was founded on evidence from Drosophila in which rearrangements of the banding pattern are readily detected on giant (polytene) chromosomes, the widespread occurrence of chromosomal rearrangements is now apparent in light of the many whole genome sequences from yeasts to humans and plants. In dipteran flies, including anopheline mosquitoes, paracentric inversions are the most common type of chromosomal change. Inversion polymorphisms often segregate in nonrandom patterns with respect to environmental and phenotypic variation, suggesting that inversions are subject to strong selection [2]–[4]. Adaptations associated with alternative arrangements can be preserved, despite migration and gene flow between their carriers, through suppressed recombination in inversion heterozygotes. This mechanism may contribute to ongoing ecotypic differentiation and ultimately speciation [5], [6], explaining why fixed chromosomal inversion differences often distinguish closely related species [4].

Given their significance in evolution, an appreciation for how inversions arise in natural populations is of great interest. Transposable elements (TEs) are known to induce chromosomal rearrangements indirectly through ectopic recombination or aberrant gene conversion and directly by an alternative transposition process [7]. Both class I elements (retrotransposons) and class II elements (transposons, including foldback elements) have been implicated in the generation of rearrangements in a wide array of organisms [2], [8], [9]. However, repetitive sequences recognizable as TEs are not inevitably associated with the breakpoints of chromosomal inversions [10]–[13]. Moreover, their occurrence at one or both breakpoints may not unambiguously implicate TEs in generating the inversion, as their arrival may have been secondary [14]. Although they constitute a proven mechanism, TEs are neither the exclusive nor perhaps the predominant means of chromosomal rearrangement.

Segmental duplications are direct or inverted repetitions of blocks of the genome from 1 to >400 kb in length and 90–100% sequence identity [15]. A growing body of evidence suggests that paralogous segmental duplications, also known as low-copy repeats (LCRs), may be important determinants of genomic plasticity in eukaryotes [16]–[18]. In nine model eukaryotes, genome-wide computational analyses of inverted repeats revealed that these structures can be quite frequent, even after masking of known repetitive elements [16], [17]. Because the arms are inverse complements of one another, inverted repeats can form secondary structures (palindromes or stem-loops) that are prone to recombination, leading to chromosomal rearrangements including inversions. This mechanism underlies several human genomic disorders, contributes to tumorigenesis, and has played a major role in primate karyotype evolution [19], [20].

**Figure 1:**
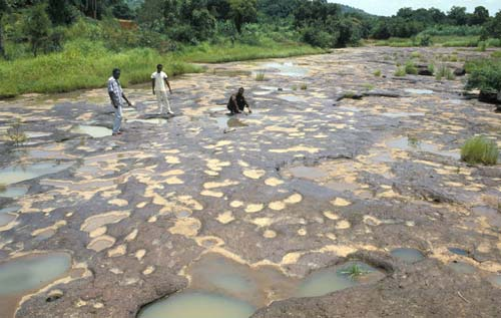
Rock pools along the Niger River, typical breeding sites of the *An. gambiae* Bamako chromosomal form. Courtesy of M. Fodde and M. Coluzzi.

*Anopheles gambiae sensu stricto*, a principal Afrotropical vector of malaria, is one of seven recognized species in the *An. gambiae* sibling species complex. Chromosomal inversions have been and continue to be instrumental in the evolutionary diversification of this group. No less than 120 polymorphic paracentric rearrangements have been described within the complex, and all except one pair of species can be distinguished by at least one fixed inversion difference [21]. *An. gambiae s.s.* (hereafter, *An. gambiae*) is one of the most chromosomally polymorphic species in the complex. Not coincidentally, it is also one of the most geographically widespread species, occupying diverse ecoclimatic zones and ecological settings across Africa south of the Sahara. Data compiled from several decades of study in West Africa, entailing nearly 18,000 karyotyped *An. gambiae*, show nonrandom associations of five inversions on the right arm of chromosome 2 that correspond to different ecophenotypes termed chromosomal forms [22], [23]. Three of these coexist in Mali–the Mopti, Savanna and Bamako chromosomal forms–each with characteristic larval breeding sites. Mopti is associated with semi-permanent sites such as rice fields, Savanna with rain-dependent sites, and Bamako with rock pools at the margins of the Niger River in southern Mali and Guinea (Figure 1). In addition to its distinctive larval ecology, Bamako is characterized by fixation of the 2*Rj* chromosomal inversion, which is absent from Mopti and present only at very low frequency in Savanna populations [24]. Other 2R inversions associated with Bamako (*b,c,* and *u*) are shared between chromosomal forms, albeit at different frequencies.

The only other inversions in the *An. gambiae* complex whose breakpoint structures have been characterized are 2*La* from *An. gambiae* and 2*Rd'* from *An. arabiensis* [12], [25]. The geographically widespread and interspecific distribution of 2*La* point to a relatively ancient origin; no decisive evidence of TEs was found at the breakpoints, though repetitive structures suggestive of their remnants were present. By contrast, inversion 2*Rd'* is exclusive to *An. arabiensis* and is apparently absent from populations east of the Rift Valley, raising the possibility of a more recent origin. A TE fragment found at the distal breakpoint designated *Odysseus* was implicated in the generation of 2*Rd'* [25]. Because 2*Rj* is limited to West African populations of *An. gambiae*, this inversion also may be relatively young. To gain insight into its origin, we cloned the 2*Rj* inversion breakpoints. Here, we describe the breakpoint structure and report the presence of giant perfectly conserved inverted repeat sequences at both junctions that are distinct from TEs. The implications of this structure are evaluated in light of genome-wide computational analysis, *in situ* hybridizations and sequencing of field specimens of *An. gambiae*.

## Results

### Isolation of the 2*Rj* breakpoints

**Figure 2:**
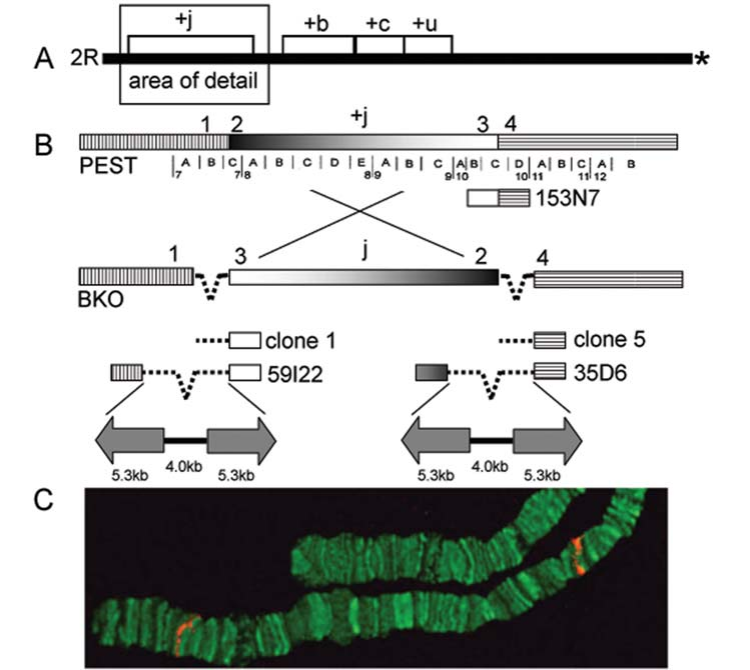
Cloning strategy for the 2*Rj* inversion breakpoints. (A) Relative position of four paracentric inversions (*j*, *b*, *c*, *u*) on chromosome 2*R* in *An. gambiae*. Asterisk represents the centromere. (B) Schematic of alternative arrangements of 2*Rj* in PEST (+^j^) and BKO (*j*) strains (not to scale). The cytogenetic map represented beneath the PEST chromosome shows the breakpoints positioned in subdivision 7C and 10C. Numbers above the chromosomes are used in the text to refer to corresponding regions adjacent to the breakpoints, represented as identically hatched or shaded regions in alternative arrangements. Dashed line indicates repetitive DNA insertion. Shown beneath the PEST chromosome is a representation of BAC 153N7 that spans the proximal breakpoint. Below the BKO chromosome are plasmid (1, 5) and fosmid (59I22, 35D6) clones corresponding to the breakpoints of the *j* arrangement. Structure of the repetitive DNA in 59I22 and 35D6 is represented below as arrows (inverted repeats) separated by a solid line (central spacer) of indicated lengths in kb. (C) FISH performed on the chromosomes of the BKO strain (2*Rj*) with BAC 153N7. Signals in red indicate the distal and the proximal breakpoints of the *j* inversion.

The *An. gambiae* reference genome, sequenced from the PEST strain, is homokaryotypic standard (uninverted) for all chromosome arrangements [26]. From a BAC library prepared from PEST [27], candidate clones known to map in the vicinity of the 2*R+^j^* proximal breakpoint (cytogenetic subdivision 10C) were hybridized to 2*Rj* chromosomes from the BKO strain of the Bamako chromosomal form of *An. gambiae*. BAC clone 153N7 hybridized to both the proximal and the distal (subdivision 7C) ends of the 2*Rj* arrangement (Figure 2), indicating that it contained inversion breakpoints. Subsequent rounds of hybridization to 2*Rj* chromosomes using smaller fragments of 153N7 refined the location of the proximal breakpoint to a 1.3 kb segment of single-copy PEST DNA. This fragment was used as a probe to isolate the corresponding sequences from a plasmid genomic library prepared from the BKO (2*Rj*) strain.

Two plasmids, clone 1 (11.3 kb) and clone 5 (11.5 kb), were recovered from the screen and completely sequenced. Each clone was characterized by a small terminal region of single-copy DNA corresponding to subdivision 10C in PEST (1.6kb and 168bp, respectively), but derived from opposite ends of the 2*Rj* inversion in BKO (Figure 2). The remaining sequences of clones 1 and 5 were repetitive and 99% identical between the two clones across their entire length. Neither clone contained sequences similar to subdivision 7C, indicating that the opposite breakpoint was not present.

To recover clones with inserts of sufficient length to span the breakpoints, a fosmid library constructed from the same BKO strain was screened by PCR, using primer sequences designed from the unique regions of clones 1 and 5. One clone from each breakpoint was isolated: fosmid 59I22 (distal) and fosmid 35D6 (proximal). Interrogating the *An. gambiae* PEST genome with end-sequences of both BKO fosmid clones revealed matches to unique regions of 7C and 10C at opposite ends, indicating that both clones spanned the long repetitive sequences at the breakpoints (Figure 2). Sequence was determined across the breakpoint junctions in both fosmids and extended 2–4 kb into flanking sequences at either end, totaling 22 kb for 59I22 and 19 kb for 35D6.

**Figure 3:**
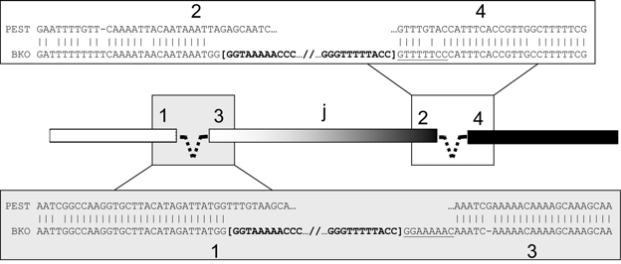
Sequences at the breakpoints of alternative arrangements of 2*Rj.* The standard arrangement represented by PEST is aligned to the inverted arrangement represented by BKO in the regions (1–4) immediately surrounding the breakpoints. Vertical lines indicate sequence identity. In bracketed bold text is the terminal sequence of the intervening inverted repeat (represented by a dashed line in the diagram). Underlined are putative target site duplications in inverse complementary orientation.

The pairwise alignment of 2*R+^j^* standard (PEST) and 2*Rj* inverted (BKO) sequences revealed the approximate nucleotide position of the inversion breakpoints (Figure 3). Spanning three divisions of the cytogenetic map (7C–10C), the 2*Rj* inversion physically encompasses 12.5 Mb between positions 3,262,186 and 15,750,717 on chromosome 2R in PEST assembly AgamP3. The distal breakpoint in 7C (between regions labeled in Figure 2 as “1” and “2” on the standard arrangement of PEST) occurred between positions 3,262,186 and 3,262,296; the intervening 110 bp are missing from the inverted chromosome. Remarkably, the proximal breakpoint in 10C (between regions “3” and “4” on the PEST chromosome in Figure 2) seems to have occurred precisely between positions 15,750,716-15,750,717.

### The 2*Rj* breakpoints contain long inverted repeats

The 2*Rj* arrangement of BKO is framed by two 14.6 kb insertions that are absent from the corresponding locations on 2*R+^j^* in PEST (Figure 2). The distal and proximal insertions are >99% identical to each other across nearly their entire lengths (14,608 identities across 14,627 aligned positions). To search for direct or inverted repeats within each insertion, the sequences were aligned against themselves in both orientations and the results were summarized by dot-plot (not shown). These analyses revealed that each insertion is composed of 5.3 kb terminal inverted repeats (IRs) separated by a 4 kb middle segment (spacer). The IRs belonging to the same insertion are >99% or 100% identical (proximal or distal insertion, respectively).

The insertions are repetitive in the *An. gambiae* genome, but not as a contiguous 14.6 kb sequence in the genome assembly. A nucleotide-level interrogation (BLASTN) of the *An. gambiae* genome using the 14.6 kb insertion sequence as a query returned >3000 hits with expectation values (E) <10^−5^, predominantly from relatively short unmapped scaffolds. However, no BLAST alignment exceeded 2kb in length, nor did multiple alignments from the same hit approximate the full-length insertion sequence, although the 118 kb unmapped scaffold AAAB01008935 bears three consecutive islands of similarity corresponding to ∼7 kb of the insertion sequence. Nevertheless, we cannot rule out that the absence of full-length hits was an artifact of large gaps (represented by strings of N's) between contigs within repetitive unmapped scaffolds. Indeed, it seems likely that automated genome assembly pipelines would have difficulty assembling a sequence with 5.3 kb perfect (or nearly perfect) IRs.

More refined BLASTN searches with smaller components of the sequence were conducted iteratively, in an effort to reconstruct consensus sequences from potential gene or TE fragments. These were followed by protein-level (BLASTX) searches at NCBI of the non-redundant GenBank database. Weak similarities (E≅10^−8^) were identified to a transposase in the IRs and to the Drosophila protein product of gene CG5555 in the spacer. Based on sequence similarity, CG5555 may have zinc ion binding and ubiquitin-protein ligase activities. However, any sequence similarities to the 14.6 kb sequence were only fragmentary and are unlikely to correspond to functional proteins. Overall, the 14.6 kb insertions lack obvious coding potential, as judged by the absence of significant open reading frames. Different parts of the insertion are highly repeated in the *An. gambiae* genome and share segments of sequence similarity with various Class I and II TEs, but are not themselves full-length elements. Screening of the insertion sequence against a reference collection of repeats (Repbase) using CENSOR software [28] identified putative fragments of *An. gambiae* TEs, including Harbinger1_AG DNA transposon in the IRs and SINEX-1_AG and GYPSY9-LTR_AG retrotransposons in the spacer (Supplementary Table S1). RepeatMasker (http://www.repeatmasker.org; [29]) a program that screens sequences for interspersed and simple repeats, returned congruent results. No association with MITEs was observed.

To avoid the implication that the 14.6 kb insertions are TEs, the full length structures have been designated as low-copy repeats (LCRs).

### Instability associated with LCRs

**Figure 4:**
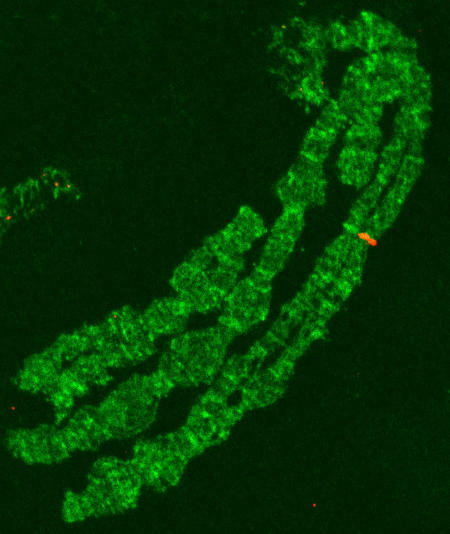
FISH performed on the chromosomes of the BKO strain (2*Rj*) with one arm of the LCR IR as a probe. Signal in red maps to subdivision 14D (on 2*R*) in only one homolog. This site also was labeled in only one homolog on the chromosomes of a wild specimen from Bancoumana, Mali.

A preliminary assessment of the presence of these LCRs (or transposon/retrotransposon components of them) at the 2*Rj* breakpoints of other *An. gambiae* isolates was performed by fluorescent *in situ* hybridization (FISH) to polytene chromosomes, using one of the IRs as a probe. As expected, signals were detected at both breakpoints in the BKO strain from which the fosmid and plasmid clones were derived. The same pattern also was detected in a wild specimen of the Bamako chromosomal form (2*Rj* homozygote), but only one of the homologs gave a signal at the proximal breakpoint (not shown). None of the 2*R+^j^* karyotypes from Mali, Burkina Faso or Cameroon were labeled at either breakpoint. Other sites of hybridization were detected in all specimens. Centric heterochromatin and the heterochromatin of subdivision 21A on 2L were weakly and diffusely labeled. Additional euchromatic sites were labeled on all arms except 2L: X (1C); 2R (14D, 16A, 18D, 19D); 3L (39A); 3R (30A, 33C). These sites were rarely occupied in more than one specimen, and sometimes appeared to be occupied only in one homolog (Figure 4). We found no more than four euchromatic sites labeled per specimen. At this level of resolution, it was not possible to determine whether the labeled sites are occupied by a complete LCR or merely related transposons of which they are composed.

**Figure 5:**
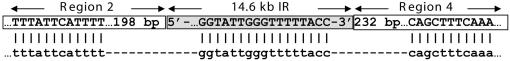
Structure of the LCR excision found at the proximal end of 2*Rj* in a subsample of the Bamako chromosomal form from Mali. Uppercase letters within boxes represent intact chromosomal regions 2 and 4, and the complete 14.6 kb LCR (shaded in gray). Shown below is the corresponding sequence after excision of the LCR. Dashes represent deleted sequence. The excision event also removed the flanking 198 bp of region 2 and 232 bp of region 4, but left 17 bp at the 3′ end of the LCR.

IR-containing sequences may be prone to deletion, as was hinted by the FISH result at the proximal inversion breakpoint in the 2*Rj* homozygote. The possibility of polymorphism for the presence of the LCR at one or both breakpoints was investigated further by PCR, using a panel of genomic DNA extracted from 249 2*Rj* homokaryotypic mosquitoes collected from Mali. Reasoning that amplification across the junction of regions 1–3 and 2–4 could only be successful if the 14.6 kb LCR had been deleted, we attempted PCR across both junctions. No products were obtained from the distal (region 1–3) junction. However, consistent with the FISH result, 70 mosquitoes (28%) yielded PCR products from the proximal (region 2–4) junction. Interestingly, the products were all identical in length and much shorter than the predicted size (184 bp instead of 606 bp) given precise deletion of the LCR. Sequence analysis of a subset (66 amplicons) revealed identical sequences, with the exception of a high frequency C–A polymorphism (SNP) at one position. The shorter than expected product size proved to be the result of an imprecise deletion that removed all except the last 17 bp of the distal end of the LCR as well as ∼200 bp of flanking DNA from both region 2 and 4 (Figure 5).

### Predicted genes immediately surrounding the breakpoints of 2*Rj*

Manual models of predicted genes nearest the breakpoints suggest that no genes were interrupted by the inversion, though this has not yet been experimentally verified. (The manual models for prospective genes in these regions can be visualized at VectorBase by navigating to the following URLs: http://agambiae.vectorbase.org/Genome/ContigView/?c = 2R:15750000;w = 20000 and http://agambiae.vectorbase.org/Genome/ContigView/?c = 2R:3262200;w = 20000, and selecting the manual annotation track available through the DAS sources menu.) The proximal breakpoint falls between two divergently transcribed genes. The first, AGAP002110-RA, potentially encodes a kinase related to the CDC7 family. The product of the second, AGAP002111-RA, contains a CCCH-type zinc finger with a G-patch domain suggesting its involvement in nucleic acid binding. These genes (henceforth, 2110 and 2111) respectively lie 6.9 kb and 407 bp from the breakpoint in regions 3 and 4 of Figure 2. The distal breakpoint (between regions 1 and 2 of Figure 2) also falls between two divergently transcribed genes, AGAP001334-RA and AGAP001335-RA (henceforth, 1334 and 1335). The potential product of 1334, 721 bp from the breakpoint, did not match any known conserved domains and has an unknown function, but was similar to mesenchymal stem cell protein DSC92. The latter gene, whose manual model is positioned only 371 bp from the breakpoint, appears to encode a dynein light chain of type-1. Sufficient sequence was determined from the BKO fosmids to align three of the four putative genes from BKO and PEST across their lengths. Gaps, if any, were limited to predicted introns and sequence identity was >96%.

### Recent origin of 2R*j*

An estimate for the minimum age of 2R*j* was derived under a model that assumes a unique origin for the inversion, complete genetic isolation of alternative arrangements, and balancing selection which has maintained the inversion at its present frequency since immediately following its origin [30]. The estimate is based on the observed number of segregating sites within the inverted and standard chromosomes at a locus inside the inversion adjacent to the breakpoint, where neither crossing over nor gene conversion are expected [31]. Using homokaryotypic (2R*j*/*j* or 2R+^j^/+^j^) mosquitoes collected in Mali, sequences were determined from two predicted genes, one inside the inversion only 6.9 kb from the proximal breakpoint (2110) and another outside the inversion only 712 bp away from the distal breakpoint (1334). The results are summarized in Table 1. Although loci outside the inversion are homosequential on chromosomes carrying alternative arrangements, diversity is considerably reduced on inverted (derived) chromosomes due to asynapsis in heterokaryotypes [31]. As expected, diversity was three-fold lower on 2Rj versus 2R+^j^ chromosomes at locus 1334 outside the inversion. Within the inversion at locus 2110, diversity was reduced nearly 19-fold on inverted versus standard chromosomes. Using the data from locus 2110, we estimated the historical frequency of 2Rj as 0.108 and the minimum age as 0.4N_e_ generations. There were no mutations shared between alternative arrangements but, consistent with a recent origin, there were also no fixed differences (Supplementary Figure S1).

**Table 1:** Levels of polymorphism at two loci near the *2Rj* breakpoint junction from standard and inverted chromosomes.

Gene^a^	No. of Chromosomes	No. ofSites^b^	S	Syn	Non	π	θ
1334 (outside inversion)							
2R+^j^	28	398	18	18	0	0.0082	0.0116
2Rj	18	397	6	5	1	0.0028	0.0044
2110 (inside inversion)							
2R+^j^	20	412	16	14	2	0.0075	0.0109
2Rj	22	410	2	2	0	0.0004	0.0013

S, number of segregating sites; Syn, number of synonymous changes; Non, number of nonsynonymous changes; π, average pairwise difference per site; θ, population mutation rate parameter 4Nμ per site based on S.

a Complete gene ID numbers are AGAP001334 and AGAP002110.

b Number of sites used in calculations. Introns and gaps were excluded.

## Discussion

Molecular analysis of both breakpoint junctions of 2*Rj* chromosomes revealed that they bear nearly identical 14.6 kb insertions that we have classified as LCRs. Each LCR is bounded by identical 5.3 kb IRs separated by a 4 kb spacer. There is no apparent coding capacity and no similar full-length structures were detected in the *An. gambiae* reference genome, not even at the *2R+^j^* breakpoint junctions. Instead, the LCRs show weak and fragmentary similarity in portions of the IRs and spacer to several unrelated class I and II TEs, suggesting their origin in a graveyard of degenerate TEs. There is a superficial structural resemblance to foldback TEs, a distinctive category of Class II TEs first discovered in *Drosophila melanogaster* [32] and later found in a wide variety of organisms. Foldback elements carry long terminal IR arms from several hundred to a thousand base pairs long separated by a spacer of variable length. They move by an unknown mechanism and most apparently lack coding capacity [33]. However, the similarity to the *An. gambiae* LCRs seems spurious for several reasons. First, the IRs of the *An. gambiae* LCR substantially exceed the 1–2 kb maximum length reported for any other foldback elements, and lack the terminal blocks of subrepeats often associated with this type of TE. Unlike foldback elements, the *An. gambiae* LCRs are not flanked by short target site duplications as expected from TE mobilization (however as discussed below, 8 bp sequences that may have been caused by an original insertion event are present). More important, no other copies could be recognized in the *An. gambiae* genome that would indicate replicative behavior typical of a foldback or any other kind of TE. Indeed, it seems unlikely that these LCRs are functional TEs or composite TEs. Available data suggest that they are segmental duplications, distinct from high copy mobile elements such as SINEs, LINEs and other TEs. The human genome is particularly enriched for segmental duplications [34], but they occur in all eukaryotic genomes [18]. Unlike transposition that results in the amplification of specific TE sequences, segmental duplications involve “generic” DNA regions of variable length (1 to >400 kb) arising from sister chromatid exchange or double-strand break repair associated with gene conversion.

Segmental duplications have been implicated in genome restructuring of primates, and have played a direct role in generating recurrent chromosomal rearrangements underlying human disease [19], [20]. Similarly, the 14.6 kb LCR is directly implicated in generating inversion 2*Rj*, given its presence at both junctions and the absence of any other repetitive structures at the breakpoints. The inversion could have originated by one of two alternative mechanisms. First, if both 14.6 kb LCRs were already present at both future breakpoints on the standard chromosome, these nearly identical sequences could serve as targets for non-allelic homologous recombination, leading to inversion of the intervening sequence. Normally, inversion is the outcome only if the non-allelic sequences are present in inverted orientation relative to one another. In the present case, because each 14.6 kb insertion is composed of 5.3 kb terminal IRs, inversion could be the outcome even if the full-length structures were present in direct orientation, via pairing of opposite IR arms. (Unfortunately, PCR experiments to determine the relative orientation of the two insertions based on the unique spacer sequence were unsuccessful).

A second possible mechanism that could have induced the inversion has been referred to as alternative or aberrant transposition in TE studies [7], [35], but this process need not be exclusive to TEs. Under this scenario, the starting point would have been a single 14.6 kb LCR at the proximal breakpoint on the standard chromosome. Synapsis between IRs on sister chromatids, followed by (1) a double strand break at one end and (2) insertion of the “hybrid” structure into a new target site (*i.e*., the other breakpoint of 2*Rj*) on one of the same chromosome arms involved in the hybrid would result in an inversion [7]. As the final outcomes of homologous recombination and alternative transposition are the same, the data do not allow us to conclusively distinguish these two mechanisms; either one is plausible. However, some evidence supports the second mechanism whereby a single proximal LCR promotes the inversion. Underlined in Figure 3 is a putative 8 bp target site duplication. This duplication originally would have existed as a short direct repeat flanking the proximal 14.6 kb LCR on the standard chromosome, and may have resulted from double-strand break repair upon its integration. As a result of the inversion, the 8 bp sequence now exists in inverse complementary orientation with each site flanking different copies of the LCR, as expected (*e.g*., Figure 3 of [36]). No reciprocal set of target site duplications was noted, probably because they were never formed during resolution of the alternative transposition process.

If the 2Rj inversion was caused by the 14.6 kb LCR, how might the LCR itself have arisen? No structure matching the full-length LCR (or its IR components) was identified in the *An. gambiae* reference genome. However, a short (118 kb) unmapped scaffold AAAB01008935 contained large adjacent segments of similarity extending consecutively across ∼7 kb of the insertion sequence. Scaffolds that are both short and unmapped are typically composed of repetitive DNA, which interferes with assembly and ability to assign a unique map location. Regions of the *An. gambiae* genome where repetitive DNA is significantly overrepresented are pericentric heterochromatin and the fully heterochromatic Y chromosome (*e.g*., [37]); such regions seem to be sinks for transposable elements and other repeat structures [38]. In these same regions of the human genome, segmental duplications are enriched 3–5 fold. It seems likely that the 14.6 kb LCR originated in one of these regions. Achaz *et al* [18] proposed a model for the dynamics of duplication in eukaryotic genomes based on computational analysis. The engine driving their model is the continuous genesis of adjacent direct (tandem) repeats created by mispairing and recombination between sister chromatids. The tandem repeats are subsequently submitted to rounds of chromosomal rearrangements (deletions, insertions, inversions) that transform them into distantly spaced repeats. According to this model, the 14.6 kb structure could have been generated by a two-step process beginning with the tandem repetition of a 5.3 kb segment (itself a TE graveyard). A later inversion event involving a 9.3 kb DNA segment containing one of the tandem repeats could have produced 5.3 kb IRs separated by a 4 kb spacer. The presence of long IRs capable of forming a stem-loop structure may have destabilized the 14.6 kb module, leading to its excision from pericentric heterochromatin and reinsertion into the proximal breakpoint of the 2*Rj* inversion.

Long IRs are associated with genetic instability. The rate with which deletions and rearrangement events are stimulated depends upon length of the IRs and the distance between them: longer IRs and shorter spacers are more recombinogenic. In yeast, 185 bp IRs separated by 8 kb spacers did not stimulate recombination, but 1 kb IRs of a perfect palindrome increased recombination as much as 17,000-fold [39]. Because the 2*Rj* inversion spans a very large distance (12.5 Mb), interaction between IRs at either end–while not impossible—should be very rare. However, interaction between the relatively long 5.3 kb IRs separated by only 4 kb within a given 14.6 kb module may be more frequent. Excision of inverted repeats is an outcome that follows intra-strand hairpin formation and slippage during DNA replication [40]. PCR and sequence data from the proximal breakpoint junction of inverted (2*Rj*) chromosomes from Mali are consistent with this model. Almost one-third of the chromosomes tested revealed an excision of the proximal 14.6 kb module. All the sequences were identical or nearly so (only one SNP was segregating), suggesting that these sequences probably descended from the same event. This event left the footprint of an imprecise excision that included flanking DNA on both sides, although a short segment (17 bp) of the 3′-end of the module was spared, consistent with slippage during DNA replication (see Figure 5). Although only one type of excision event was detected and only at the proximal breakpoint, additional events could be unmasked with wider geographic sampling and/or a wider selection of PCR primers for screening.

Like most mosquitoes, *An. gambiae* possesses only 3 chromosomes: two autosomes and sex chromosomes. Accordingly, any randomly occurring inversion has a reasonable chance of capturing a significant fraction of the genome, potentially including sets of genes relevant to adaptive fitness in alternative environments. However, a striking pattern found in *An. gambiae* is the overrepresentation of polymorphic inversions on chromosome 2R. Although this arm represents <30% of the polytene complement, it carries 58% of all polymorphic inversions [21]. Rearrangements of this arm are associated with ecotypic differentiation and incipient speciation of *An. gambiae* [21], [41]. Therefore, both the origin and the gene content of these inversions are of special interest in an evolutionary and epidemiological context: ecotypic differentiation is increasing the vectorial capacity of this already proficient carrier of malaria [21]. Here we have shed light on the origin of the 2*Rj* inversion that is characteristic of the Bamako chromosomal form of *An. gambiae*. This 12.5 Mb inversion was not induced by TEs *per se*, but rather by almost identical long IR modules at both breakpoints that are genetically unstable LCRs. Sequence identity of >99% between both IR arms within a LCR, as well as between the two LCRs, suggests either the homogenizing effect of gene conversion or the relatively recent origin of the 2*Rj* inversion. A recent origin is consistent with our estimates of the minimum age of the 2Rj inversion based on sequence polymorphism near a breakpoint (∼0.4Ne generations) and the lack of fixed differences between alternative arrangements. Three other considerations support the hypothesis of recent origin. First is the absence of any other repetitive DNA at the breakpoints, despite the expectation that IRs at inversion breakpoints should be mutational hotspots for subsequent structural and nucleotide variation [42]. Second is the apparent absence of these modules from the *An. gambiae* reference genome. Third is the restricted distribution of 2*Rj* in Africa. *An. gambiae* occurs throughout tropical Africa, but 2*Rj* is not found outside of West-Central Africa and the Bamako chromosomal form that is fixed for this inversion is limited to the Niger River and its tributaries in southern Mali and northern Guinea [22]. If this rearrangement was indeed recent, the expected sequence conservation at the breakpoints of inverted chromosomes should facilitate ongoing efforts to design a robust molecular diagnostic assay to detect the inversion in natural populations. Successful development of a molecular diagnostic assay will facilitate future studies on the ecological genomics of 2*Rj*, as current efforts to study ecological and epidemiological correlates associated with this inversion are severely hampered by limited access to readable polytene chromosomes.

## Materials and Methods

### Mosquitoes

The BKO colony of the *An. gambiae* Bamako chromosomal form used for genomic library construction originated from Moribabougou, Mali in 1998. It was established at the University of Rome “La Sapienza” and kindly provided by A. della Torre. The karyotype was 2*Rjcu*/*jcu*. Field specimens of *An. gambiae* Bamako were collected in Aug-Sep 2004 at four sites in Mali: Bancoumana (12°20′N, 8°20′W), Banambani (12°48′N, 08°03′W), Fanzana (13°20′N, 06°13′W) and Kela (11°88′N, 8°45′W).

### FISH

Ovaries dissected from half gravid females were placed in modified Carnoy's solution (ethanol: glacial acetic acid, 3∶1) for 24 h at RT. Polytene chromosome preparation and *in situ* hybridization were performed according to [43], [44] as described previously [12]. Following hybridization, chromosomes were washed with 0.2×SSC, counterstained with YOYO-1 (Sigma) and mounted in DABCO (Sigma) antifade solution. Fluorescent signals were detected using a Bio-Rad MRC-1024 Confocal Laser Scanning Imaging System running LaserSharp 3.2 software.

### Library Construction and Screening

High molecular weight genomic DNA extracted from the *An. gambiae* BKO strain (Qiagen Genomic-tip) was used to prepare plasmid (pSMART LCKan cloning vector) and fosmid (pCCFos cloning vector) libraries (Lucigen Corporation, Middleton, WI). Three genome-equivalents of the plasmid library (average insert size, 12–15 kb) were transferred to nitrocellulose supported membranes (Nitropure, GE Osmonics) and screened according to the manufacturer's recommended protocol. The probe was a 1.3 kb fragment of a BAC clone (153N7) derived from the *An. gambiae* PEST (2R+^j^/+^j^) strain by PCR using primer A (5′-GTTGACGTTTTGGGTCGTTT-3′) and primer C (5′-CTCGGTTTGGGAAAAAGTCA-3′). Labeling was by PCR in the presence of ^32^P-dCTP. The 50 µl PCR reaction contained 20mM Tris-HCl (pH 8.4), 50 mM KCl, 1.5 mM of MgCl_2_, 200 µM of each dNTP excluding dCTP, 50 µCi of ^32^P-dCTP (Amersham), 50 ng of 153N7 template, 25 pmol primers, and 1U Taq polymerase. Following an initial 4 min denaturation at 94° were 35 cycles of 94° for 1 min, 60° for 15 s, 72° for 4 min and a hold at 72° for 5 min. The probe was purified using a Centri-Sep column (Princeton Separations) as recommended by the supplier.

The *An. gambiae* BKO fosmid library (average insert size ∼40 kb) was arrayed onto 120 384-well plates, representing ∼10 genome equivalents. Screening was achieved by PCR amplification of DNA pools (1-plate pools, 8-row and 12-column DNA pools per each plate). Primers were designed from the unique regions of two clones (1 and 5) isolated from the plasmid library, derived from the distal and proximal breakpoints of the 2*Rj* inversion (see Figure 2). Primers for the distal breakpoint were BfosF1 (5′-AAGCAAGACGCCGAGATTGC-3′) and BfosR1 (5′-TTTTCCAAACAATCGCGTGC-3′); those for the proximal breakpoint were BfosF5_2 (5′-TTCCCATTTCACCGTTGCCT-3′) and BfosR5_2 (5′-CATTATGCATTTCTGTTGCA-3′). Each 25 µl PCR reaction contained 20mM Tris-HCl (pH 8.4), 50 mM KCl, 1.5 mM MgCl_2_, 200 µM each dNTP, 5 pmol of each primer, 1 µl of the template DNA, and 1U of Taq polymerase. The PCR conditions were 94° for 2 min, 30 cycles of 94° for 30 s, 50° for 30 s and 72° for 30 s followed by 72° for 5 min and a 4° hold.

### DNA Isolation and Sequencing

Plasmid DNA was isolated using a standard alkaline lysis protocol. Fosmid and BAC DNA was isolated using the Perfectprep Plasmid Midi Kit (Eppendorf). The sequencing strategy entailed a combination of primer walking using custom primers (Invitrogen) and the GPS-1 Genome Priming System (New England BioLabs), which places a transposon containing universal priming sites into target DNA at random locations. Sequencing reactions were performed with the Big Dye Terminator v3.1 Cycle Sequencing kit (Applied Biosystems) and analyzed on the ABI PRISM 3700 DNA Analyzer (Applied Biosystems). Individual sequences were inspected, verified and assembled into contigs using Lasergene Seqman software (DNASTAR). The sequenced regions of fosmids 35D6 and 59I22 were submitted to GenBank (accession nos. EF015880-EF015881).

### Analysis of Excision Events

Genomic DNA was isolated from individual 2*Rj*/*j* mosquitoes using the DNeasy kit (Qiagen) with the slight modification of reducing elution volume to 30 µl. Prior to analysis by PCR, genomic DNA was diluted 1∶8 in TE buffer (∼5 ng/ul). PCR across the junctions of the inverted arrangement was attempted with a pair of distal primers (D1F, 5′-GCGTTGTCAATAATGCCTGA-3′; P3ST, 5′-CGAAGAGGAAGTCGTGCTTT-3′), and a pair of proximal primers (D2ST, 5′-GGCGGATTCTAGCAAATGTC-3′; P4U, 5′-GTTGACGTTTTGGGTCGTTT-3′). Each 50 µl PCR reaction contained 20mM Tris-HCl (pH 8.4), 50 mM KCl, 1.5 mM MgCl_2_, 200 µM each dNTP, 5 pmol of each primer, 1 µl of template DNA, and 1U of Taq polymerase. The PCR conditions were 94° for 2 min, 35 cycles of 94° for 30 s, 55° for 30 s and 72° for 45 s followed by 72° for 5 min and a 4° hold. PCR products were separated on 1.5% agarose gels, excised, and purified using the Pure-Link Gel Extraction Kit (Invitrogen). PCR products were sequenced on both strands using Big Dye Terminator chemistry as described above.

### Molecular dating of 2Rj

Primer pairs for AGAP002110 (temp10F, 5′-CGGGAGGGTGTAATGGTATG-3′ and temp10R, 5′-AGAAAATTGCTCGGCTTCAC) and AGAP001334 (temp54F, 5′-TTCCCATGCAATTCTGTTCA-3′ and temp54R, 5′-TTTGCCAGTTTTCCTTCACC-3′) were used in 50 µl PCR reactions containing 20mM Tris-HCl (pH 8.4), 50 mM KCl, 1.5 mM MgCl_2_, 200 µM each dNTP, 5 pmol of each primer, 1 µl of template DNA, and 1U of Taq polymerase. The PCR conditions were 94° for 2 min, 40 cycles of 94° for 20 s, 55° for 15 s and 72° for 20 s followed by 72° for 5 min and a 4° hold. PCR products were purified and sequenced directly as described above. Sequence polymorphism statistics in Table 1 were calculated using DnaSP 4.0 software [45]. Date of origin was estimated from the observed number of segregating sites within inverted and standard chromosomes as described [30].

## Supporting Information

Table S1 CENSOR results of known transposons and retrotransposons in REPBASE to which similarity was found in the 14.6 kb Low Copy Repeat.
(0.04 MB DOC)

Figure S1 Polymorphic sites at AGAP002110 in *A. gambiae* field specimens homokaryotypic for 2*Rj*.
(0.03 MB DOC)
