# Supplementary material for: Segmental Duplication Implicated in the Genesis of Inversion 2Rj of Anopheles gambiae
Source: PLoS One. 2007 Sep 5;2(9):e849. doi: 10.1371/journal.pone.0000849 (PMC1952172; doi:10.1371/journal.pone.0000849)
Supplement: Table S1 — CENSOR results of known transposons and retrotransposons in REPBASE to which similarity was found in the 14.6 kb Low Copy Repeat. (0.04 MB DOC) [file pone.0000849.s001.doc]

Supplementary Table S1. CENSOR results of known transposons and retrotransposons in REPBASE to which similarity was found in the 14.6 kb Low Copy Repeat (14.6 kb LCR) flanking the 2Rj inversion of *A. gambiae*

| Position of match in  14.6 kb LCR: | |  | Position of match in (retro)transposon: | |  |  |  |  |
| --- | --- | --- | --- | --- | --- | --- | --- | --- |
| From | To | (Retro)transposon | From | To | Class | Dir | Sim | Score |
| 1163 | 1279 | P1_AG | 3779 | 3886 | DNA/P | c | 0.73 | 335 |
| 1288 | 1357 | hAT-2_AG | 2143 | 2212 | DNA/hAT | c | 0.74 | 291 |
| 2244 | 2419 | HARBINGER1_AG | 3484 | 3650 | DNA/Harbinger | d | 0.73 | 554 |
| 6691 | 6793 | MARINER36_CB | 1373 | 1490 | DNA/Mariner | d | 0.74 | 213 |
| 7044 | 7140 | SINEX-1_AG | 31 | 124 | Non-LTR/SINE | d | 0.86 | 535 |
| 7170 | 7400 | GYPSY9-LTR_AG | 390 | 625 | LTR/Gypsy | c | 0.73 | 768 |
| 7949 | 7979 | GYPSY9-LTR_AG | 151 | 181 | LTR/Gypsy | c | 0.90 | 247 |
| 7997 | 8065 | GYPSY15-LTR_AG | 3 | 71 | LTR/Gypsy | c | 0.81 | 378 |
| 12204 | 12379 | HARBINGER1_AG | 3484 | 3650 | DNA/Harbinger | c | 0.72 | 554 |
| 13266 | 13335 | hAT-2_AG | 2143 | 2212 | DNA/hAT | d | 0.74 | 291 |
| 13344 | 13460 | P1_AG | 3779 | 3886 | DNA/P | d | 0.74 | 335 |

Class, category and family of (retro)transposon; Dir, orientation of match to 14.6 kb LCR (c: complementary; d: direct); Sim, percent similarity; Score, alignment score from blast.
