# Supplementary material for: Segmental Duplication Implicated in the Genesis of Inversion 2Rj of Anopheles gambiae
Source: PLoS One. 2007 Sep 5;2(9):e849. doi: 10.1371/journal.pone.0000849 (PMC1952172; doi:10.1371/journal.pone.0000849)
Supplement: Figure S1 — Polymorphic sites at AGAP002110 in A. gambiae field specimens homokaryotypic for 2Rj. (0.03 MB DOC) [file pone.0000849.s002.doc]

1111111111111111111111

5555555555555555555555

7777777777777777777777

4444444444444444444444

3333333333333333333333

1111222222233344444555

7789122255900525569077

3873004728703183978128

BC19 -CTCTCCCACCGCCCGGGCGGT

BC20 -.................SR..

BC37 -.....................

BC79 -.....................

BC83 -.....................

BN16 -.....................

FZ2 -.....................

KL272 -.....................

KL371 -.....................

KL648 -.....................

KL669 -.....................

BC71 A...AM...............C

BC97 -..MA..S.YY.Y.Y......C

BN15 AT........Y..........Y

BN26 A...A...R...Y..SKS...C

FZ49 -...A.YSRYY..M.......C

FZ51 A...AM...............C

KL176 A...A.......Y..SKS..RC

MB80 A...AM......Y..S.....C

NG14 A.Y.A.....YR.M.......C

NG48 A...A.......Y........C

Numbering of polymorphic sites corresponds to the position on chromosome 2R in the *A. gambiae* reference genome AgamP3 assembly. Nucleotides identical to the first sequence are indicated by a dot. Dashes represent deletions. R, Y, K, M and S are standard single-letter c**odes of the International Union of Biochemistry (IUB)**.
